# Unveiling the Digital Phenotype of Physical Activity Behavior in Community-Dwelling Older Adults Using Machine Learning

**DOI:** 10.3390/bioengineering13020205

**Published:** 2026-02-11

**Authors:** Anas Abdulghani, Kim Daniels, Bruno Bonnechère

**Affiliations:** 1 Technology-Supported and Data-Driven Rehabiltitation, Data Sciences Institute, Hasselt University, 3590 Diepenbeek, Belgium; anasnazar4444@gmail.com; 2 Department of PXL, Healthcare, PXL University of Applied Sciences and Arts, 3500 Hasselt, Belgium; kim.daniels@pxl.be; 3 REVAL Rehabilitation Research Center, Faculty of Rehabilitation Sciences, Hasselt University, 3590 Diepenbeek, Belgium

**Keywords:** digital phenotyping, physical activity, older adults, machine learning, time series forecasting, LightGBM, recurrent neural networks

## Abstract

Physical activity (PA) is an important factor for maintaining health and well-being, especially in older adults. This study aims to apply machine learning methods to predict PA patterns and identify key factors influencing these behaviors among community-dwelling older adults. Linear and Logistic Regression, Elastic Net, and Light Gradient Boosting Machine (LightGBM) models were used to analyze cross-sectional data. While longitudinal data collected over 14 days were analyzed using LightGBM, Gated Recurrent Unit (GRU), and Long Short-Term Memory (LSTM). The most important predictors identified in the cross-sectional analysis were the Exercise Self-efficacy Scale (ESES) for PA levels and the Geriatric Depression Scale (GDS) for the International Physical Activity Questionnaire (IPAQ) as a continuous measurement. In the longitudinal analysis, using a seven-day sequence of step count data provided the best performance for forecasting physical activity for the entire next day. Overall, the findings indicate that combining wearable sensor data with machine learning and deep learning methods can provide valuable insights into physical activity behaviors among older adults. In the cross-sectional analysis, psychological and motivational factors such as self-efficacy were identified as important factors for activity levels, while in the longitudinal analysis, using a week of past step count data provided the most reliable predictions of future-day physical activity.

## 1. Introduction

According to the World Health Organization (WHO), the world population aged over 60 years will have doubled in number by 2050, with an estimated total of 2 billion people [1]. Aging is associated with some physiological alterations, with reduced aerobic capacity (indicated by declining maximal oxygen consumption or ${\mathrm{VO}}_{2\max}$ in inactive individuals) and sarcopenia (loss of skeletal muscle mass and strength). These are crucial with respect to quality of life, functional independence, and mortality. Physical inactivity can exacerbate these conditions [2]. Physical activity (PA) is defined as any bodily movement produced by skeletal muscles that requires energy use (measured in kilocalories). Exercise is a subset of PA that is deliberate, organized, and repeated, with the aim of enhancing or maintaining physical fitness [3]. Since older adults spend more time in low-intensity PA than doing exercises, self-report approaches to PA can suffer from recall and response bias [4]. As a result, objective and passive wearable monitoring is a better method to track moment-to-moment PA than self-reports [5].

According to the WHO guidelines, older adults should engage in 150–300 min of moderate-intensity aerobic activity per week, or 75–150 min of vigorous-intensity aerobic activity, or a comparable combination of both. They should also participate in muscle-strengthening exercises at moderate or higher intensity for all major muscle groups at least twice per week. Furthermore, multicomponent physical activity, which focuses on functional balance and strength training, should be included on three or more days weekly at moderate or greater intensity. However, many older adults do not adhere to these recommendations [6]. Specifically, according to the World Health Survey, 18.80% of men and 24.50% of women aged 60–69 years, and 42.10% of men and 54.60% of women aged 80 years or older, did not meet the minimum aerobic physical activity guidelines [7]. This can be associated with a rise in noncommunicable diseases such as cardiovascular diseases, type 2 diabetes, stroke, and dementia [8]. Regular PA in older adults is associated with some health benefits. This includes improvements in physical function and enhanced mental and cognitive well-being [8]. Also, some longitudinal studies indicate that PA is associated with a lower likelihood of developing dementia, notably for Alzheimer’s disease [2].

Assistive technology for older adults has achieved substantial achievements in the development of rehabilitative, adaptive, and assistive devices. This technology can offer notable support for individuals with physical impairments to live more independently, especially in terms of mobility. Older adults may rely on assistive technologies to enhance their well-being, health, and reduce their reliance on others [9].

Digital phenotyping is an emerging approach to health data collection that uses digital tools like smartphones and wearables to passively and continuously monitor physiological, behavioral, and psychological metrics. Digital phenotyping relies less on self-reports, which may help limit biases related to recall and social desirability [5]. In addition, it enables frequent tracking of PA in everyday settings, providing rich longitudinal data with repeated observations within the same individuals. These measurements reflect PA patterns that are context-dependent and may detect small changes that are difficult to capture using self-report. Furthermore, repeated measures support analyses of within-individual variability and changes over time, which help the development of models that predict longitudinal PA patterns [10]. Digital phenotyping also has the potential for early intervention and prevention of serious medical conditions. For example, longitudinal tracking of outcomes such as mobility and mood can help to detect health decline signals and enables early intervention [5].

Machine learning has supported PA research to support activity monitoring and personalized health intervention, including estimating activity levels, identifying adherence patterns, and delivering tailored feedback [11,12]. Deep learning- and machine learning-driven digital phenotyping methods offer promising new ways to capture within- and between-subject variation in PA. This includes potentially nonlinear relationships that simpler models may fail to capture [13].

This study used two datasets: a cross-sectional baseline dataset and a longitudinal wearable dataset. Physical activity was the primary outcome, measured by the International Physical Activity Questionnaire (IPAQ, as a category and a continuous score) in the cross-sectional data and by step counts using wearable devices in the longitudinal data. Mild depression measured using the Geriatric Depression Scale (GDS) was included as a secondary outcome since depression is clinically relevant in older adults, and evidence indicates that exercise interventions can prevent depression and reduce depressive symptoms in this population [14]. Furthermore, risk of falling, determined by the incidence of falling during the previous six months, was also considered as a secondary outcome because of its importance in older adults. Because falling is a contributor to morbidity in this age group [15].

Previous studies using cross-sectional designs have investigated associations between PA and demographic, psychological, and clinical characteristics [16]. However, these studies were conducted in adults in general and in cancer patients in China. Other evidence suggests that factors influencing PA may vary across cultural, age, and ethnic groups. This implies that determinants of PA can differ depending on the population under study [17]. However, evidence in community-dwelling older adults in Belgium remains limited. For this reason, it is important to examine PA predictors specifically in community-dwelling older adults in Belgium using data collected through self-reported measures and clinical examinations. Furthermore, other work has focused on forecasting short-term PA (total daily step counts) using past wearable-derived activity behaviour [18]. However, the minimum history length required to make reliable short-term PA forecasts, particularly in older adults, remains underexplored. Identifying this minimum is important to ensure that predictions are based on sufficient past PA data while also reducing participant burden. In addition, relying only on aggregated daily step counts may overlook meaningful within-day variation in activity patterns. Therefore, longitudinal analyses are needed to evaluate the minimum time window required for reliable PA forecasting in older adults, using four time segments within a day (morning, noon, afternoon, and evening). It is also important to explore whether certain time periods provide more informative input when predicting PA either within the same time segment or across different segments. For example, step counts in the morning could be predicted using past morning PA or activity from other time periods. It can also identify time periods in which PA is less predictable, suggesting that activity during these periods may be more influenced by context or external factors than by previous activity patterns. Addressing these gaps may contribute to the development of monitoring approaches that are feasible for older adults, account for within-day variation in PA behaviour, and support more targeted interventions.

These gaps are addressed in this study using two independent analyses. The first objective is to evaluate cross-sectional predictive models to identify factors associated with physical activity and related outcomes (mild depression status and fall risk) using demographic, psychological, and clinical characteristics. This is specific for community dwelling older adults in a Belgian setting. The second objective is to evaluate longitudinal forecasting models using wearable-derived step counts. Specifically, the second objective determines the minimum history length required for reliable short-term prediction. It also explores forecasting performance across different within-day time segments. It further examines whether baseline demographic, psychological, and clinical characteristics are linked to between-individual differences in forecasting performance.

The paper is structured as follows. Section 2 of the paper presents the materials and methods, including the study design, datasets, preprocessing steps, and the predictive modeling approaches used for the cross-sectional and longitudinal analyses. Section 3 reports the results, including model performance and the main predictors identified for physical activity and the related outcomes, as well as the longitudinal forecasting performance across different time windows and within-day time segments. Section 4 discusses the findings in relation to previous research, highlights methodological considerations and study limitations, and outlines implications for future work. Section 5 concludes the paper and summarizes the key contributions.

## 2. Materials and Methods

### 2.1. Study Design and Participants

Data used in the study were collected as part of a two-week prospective observational design to collect detailed information on PA behaviors and their influencing factors. The cross-sectional part involved self-reported questionnaires to collect demographic and contextual data, as well as clinical tests to assess relevant health and functional status. In addition, longitudinal data containing step counts were collected through continuous monitoring using Garmin wearable devices. The study was carried out in a natural setting to ensure that the participants could carry out their usual daily activities without interruption. A complete study description is presented in Appendix A. The inclusion criteria included participants who lived in the community, were 65 years or older, could give informed consent, were fluent in Dutch, and had no severe illness. Participants with neurological, recent cardiovascular, severe respiratory, or severe cognitive disorders were excluded. Participants were enrolled in local community services using social media exposure and newspaper ads. The enrollment started in October 2023, and the collection of data began in March 2024 [19,20]. No prior studies that were sufficiently comparable in relation to the study population and measurement approach to give information needed for sample size calculation. Therefore, performing a sample size calculation was not possible. A convenience sample of at least 100 community-dwelling older adults was chosen. In total, 108 participants were included.

### 2.2. Data Description

To collect the cross-sectional data, participants were asked to fill out questionnaires and to participate in clinical evaluations. The collected variables covered sociodemographic information, psychological scales, physical activity, clinical measures, lifestyle factors, mobility and physical capability measures, and digital health readiness. In total, data from 108 participants were collected, with 308 variables systematically recorded per participant, providing a rich multidimensional dataset capturing the physical, psychological, and clinical measures. Because PA can be associated with factors across different domains [21]. To identify the predictive factors, several methods of handling high-dimensionality using feature selection procedures and regularized modeling approaches were utilized [22,23,24].

As for the longitudinal data, participants’ daily PA (step counts) were continuously recorded over a 14-day period using the Garmin Vivosmart 5^®^ activity tracker (Garmin International, Olathe, KS, USA). Each participant had 56 time points (4 timesteps per day over 14 days), which corresponds to three-hour segments (e.g., 8:00–11:00, 12:00–15:00, 15:00–18:00, and 18:00–23:00). At each time segment or timestep, the number of steps was aggregated.

### 2.3. Data Preprocessing

Variables exhibiting very low or near-zero variance, characterized by having the same value in the majority of observations, were excluded from the analysis. This step was taken because such variables generally contribute little to predictive performance and can potentially create problems during model training [24].

The longitudinal dataset captured within-subject temporal variation in PA, with a focus on predicting the number of steps in the following day and finding the minimal time window for reliable predictions. In the longitudinal dataset, some participants had measurements for only a few days with large gaps between them, resulting in a high proportion of missing data. These participants were excluded from the analysis to ensure data completeness. Specifically, participants with more than 30% missing values in the outcome variable and without complete measurements over the 14-day period were removed. For those with more than 14 days of data, only the first 14 days were used to allow for a fair comparison. After applying these criteria, a total of 100 participants were included in the analysis. Missingness was evaluated descriptively, and no systematic association with age or other key participant characteristics was observed.

### 2.4. Predictive Modeling for the Cross-Sectional Data

In the cross-sectional analysis, each participant contributed one record, so all models (including Linear/Logistic regression) were fitted on independent observations.

Linear and Logistic Regression models were used to predict four outcomes in the cross-sectional dataset. Risk of falling, GDS category (mild depression status), and IPAQ category were binary outcomes, while IPAQ MET-minutes/week (Metabolic Equivalents of Task minutes per week) was a continuous outcome. Thus, a Linear Regression model was used for predicting the continuous outcome, while the binary outcomes were predicted using Logistic Regression models [25].

Given the large number of predictors, an information-gain ranking was used, and the 30 most informative predictors were retained for model fitting to ensure model stability. As for variables with high pairwise correlations of 60% or more, only one was selected while the others were excluded from the analysis [22].

In addition, Elastic Net models were utilized to predict the outcomes. It is a regularization and variable selection technique that can overcome some of the challenges encountered by traditional penalized regression methods, especially in high-dimensional settings where the number of predictors exceeds the number of observations. It is particularly suitable when predictors are highly correlated [23].

Furthermore, Light Gradient Boosting Machine (LightGBM, also abbreviated as LGBM) was applied. It is a gradient boosting framework that uses tree-based learning algorithms designed for efficient training, particularly suitable for complex structured data, such as the cross-sectional dataset. It employs a leaf-wise tree growth strategy with depth constraints, which often leads to improved performance compared to other methods [26].

To train and evaluate the models, the cross-sectional dataset was split into training (70%) and testing (30%) subsets using a stratified approach to maintain class balance. Stratified k-fold cross-validation (CV) was applied to the training data for hyperparameter tuning and model selection, ensuring consistent class proportions across folds. This procedure helped to reduce overfitting [27].

For model comparison, three models were fitted separately for the binary and continuous outcomes to enable comparison of their predictive performance using appropriate evaluation metrics. The choice of metrics depended on the distribution and type of each outcome variable. The Median Absolute Error (MedAE) was the main metric used to evaluate the regression models, as it provides a more robust assessment of performance than the Mean Absolute Error (MAE), particularly when the outcome distribution has outlying observations [28]. To account for differences in data scale, the MAE was divided by the mean and the MedAE by the median of the test data (MAE/Mean and MedAE/Median), providing scale-invariant performance measures.

The metrics that were utilized in the classification were Recall (Sensitivity), Specificity, Precision, Accuracy, Balanced Accuracy, F1 score, and the Area Under the Precision-Recall Curve (PR AUC) [29,30]. PR AUC was chosen as the primary evaluation metric, as it provides a more informative assessment of performance on imbalanced datasets compared with the Area Under the Receiver Operating Characteristic Curve (ROC AUC) [31].

To support the interpretation of the predictive models in the cross-sectional analysis, SHapley Additive exPlanations (SHAP) was used. SHAP is an approach that explains the contribution of each predictor to the prediction of the model for each participant. These contributions are quantified using SHAP values, where each predictor receives a numeric measure indicating its influence on an individual prediction. The SHAP values can be summarized for each predictor across participants to give the most influential predictors overall [32,33].

### 2.5. Predictive Modeling for the Longitudinal Data

Recurrent Neural Networks (RNNs) are a type of Artificial Neural Network (ANN) designed to model sequential data, making them suitable for forecasting physical activity patterns over time. To effectively capture long-term temporal dependencies, RNN architectures such as Long Short-Term Memory (LSTM) and Gated Recurrent Unit (GRU) networks were used [34].

In addition, LightGBM was applied to forecast the step counts in the longitudinal dataset by incorporating lagged values of the outcome variable as input features. In this setup, step counts from previous time points were included as predictors to estimate the number of steps at the subsequent timestep. This feature-engineering approach allows the model to capture short-term temporal dependencies in the data [35]. The participants in the longitudinal dataset were randomly divided into training (70%), validation (10%), and testing (20%) sets.

To determine the minimum number of days needed as input to predict the physical activity for the following day (consisting of 4 timesteps), the predictive performance of several model configurations for forecasting step count was compared. Therefore, three models were tested and compared: LSTM, GRU, and LightGBM.

The MAE, MedAE, and MedAE divided by the median of the test data (MedAE/Median) were used as evaluation metrics. The MedAE/Median was the primary metric because it is scale-invariant, accounting for the scale of the data, and lower values indicate better model performance.

An additional evaluation was performed to assess model performance at the participant level. Predictions for each participant were evaluated using a proportion error, calculated as:

(1) $$\begin{array}{c} \mathrm{proportion}\;\mathrm{error}\;\mathrm{at}\;\mathrm{time}\;i=\left\{\begin{array}{cc} \frac{|{\hat{y}}_{i}-y_{i}|}{y_{i}}, & \mathrm{if}\;y_{i}\neq 0\\ \frac{|{\hat{y}}_{i}-y_{i}|}{1}, & \mathrm{if}\;y_{i}=0 \end{array}\right. \end{array}$$

where ${\hat{y}}_{i}$ was the predicted value at timestep *i* and $y_{i}$ was the actual value at the same timestep. If the actual value was zero, the denominator was set to 1 to avoid division by 0. A single prediction at timestep *i* was considered correct if this proportion error was less than or equal to 0.10. A successful prediction for a particular participant was then defined as having at least 0.80 of their predicted values with proportion errors of 0.10 or less.

To assess whether participant baseline characteristics are associated with meeting the predefined success criterion, univariate comparisons were conducted between participants who did and did not meet the criterion. Wilcoxon rank-sum tests were used for continuous variables (age and IPAQ MET-minutes/week), and Fisher’s exact tests were utilized for categorical variables [36,37]. A significance level of 0.05 was applied.

**Figure 1:**
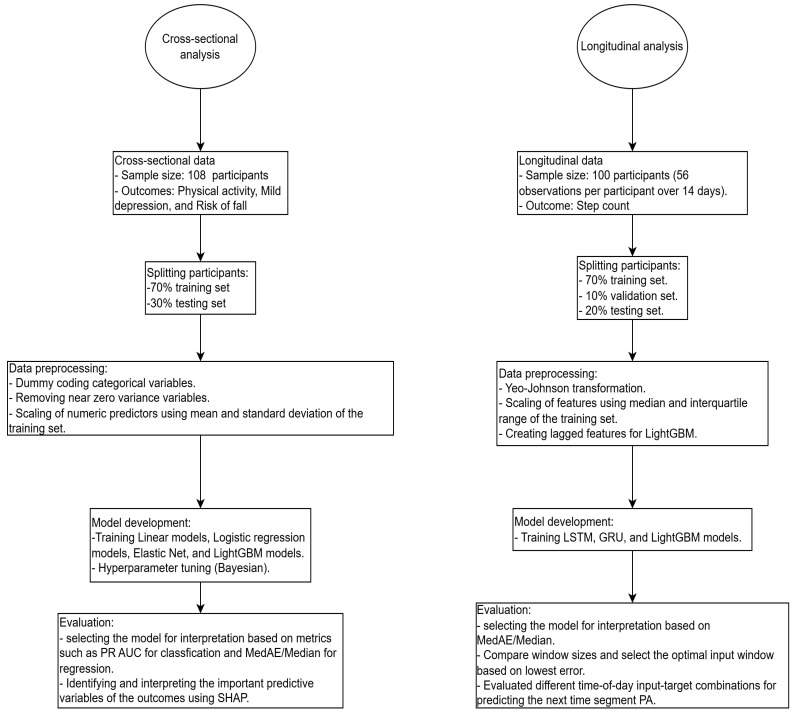
Flowchart for data processing, model development, and evaluation for the cross-sectional and longitudinal analyses.

Figure 1 shows the flowchart for the modeling of both the cross-sectional and longitudinal analyses.

## 3. Results

### 3.1. Cross-Sectional Analysis

Table 1 presents summary statistics for the continuous and categorical variables in the cross-sectional dataset. Continuous variables are reported as (SD, standard deviation) or median [P25, 25th percentile; P75, 75th percentile] depending on their distribution. The mean age of participants was 70.10 years (SD = 4.59), and the median Body Mass Index (BMI) was 26.30 [23.00; 28.40]. Participants reported a median physical activity level of 5143.50 MET-minutes/week [2642.00; 9973.30].

Among the categorical variables, most participants were married (72.20%), living with a partner (78.70%), and retired (97.20%). According to the IPAQ classification, 71.30% of participants were highly active, and only one participant was categorized as having low physical activity. Because this group was underrepresented, it was excluded from the analysis. The classification task was therefore simplified to a binary outcome, comparing moderate (negative class) and high (positive class) activity levels. Additionally, 16.70% of participants reported a fall within the six months prior to data collection, and 33.30% were identified as mildly depressed according to the GDS.

As shown in Table 2, LightGBM outperformed Logistic Regression and Elastic Net across all binary classification tasks, achieving the highest PR AUC values of 0.800 (GDS), 0.381 (risk of falling), and 0.809 (IPAQ).

For mild depression status, LightGBM achieved a Balanced Accuracy of 0.682, indicating moderate discriminative ability. Model performance was limited for risk of falling prediction (PR AUC = 0.381, Balanced Accuracy = 0.542), reflecting the effect of class imbalance.

Resampling strategies were explored to address the class imbalance, but they did not lead to meaningful improvements in performance. Applying class weights resulted in slightly better performance, but it remained insufficient for reliable fall-risk classification. For this reason, an anomaly detection approach is considered as an alternative.

As for physical activity classification (IPAQ), LightGBM achieved high Recall (0.875) and Precision (0.808), demonstrating reliable detection of highly active participants. Although the Specificity was lower (0.444), highlighting the difficulty in identifying the moderate-activity group.

Table 3 summarizes the regression results for IPAQ MET-minutes/week. LightGBM yielded the lowest normalized median absolute error (MedAE/Median = 0.551), outperforming Linear Regression (0.859) and Elastic Net (0.785).

**Figure 2:**
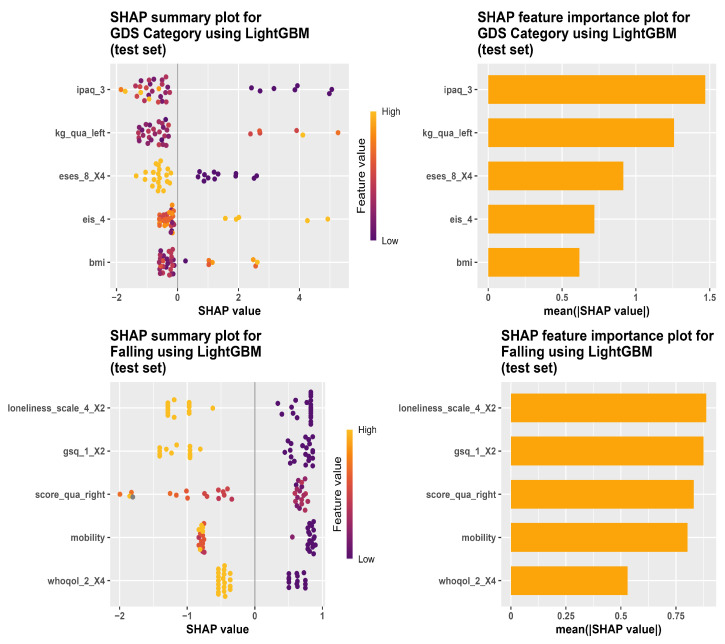
SHAP summary plots (**left**) and feature importance plots (**right**) for LightGBM models on the test set. In the SHAP summary plots, each dot represents one participant, the color indicates the feature value (low to high), and the x-axis shows the SHAP value. The top row shows the results for the GDS category, and the bottom row shows the results for falling.

**Figure 3:**
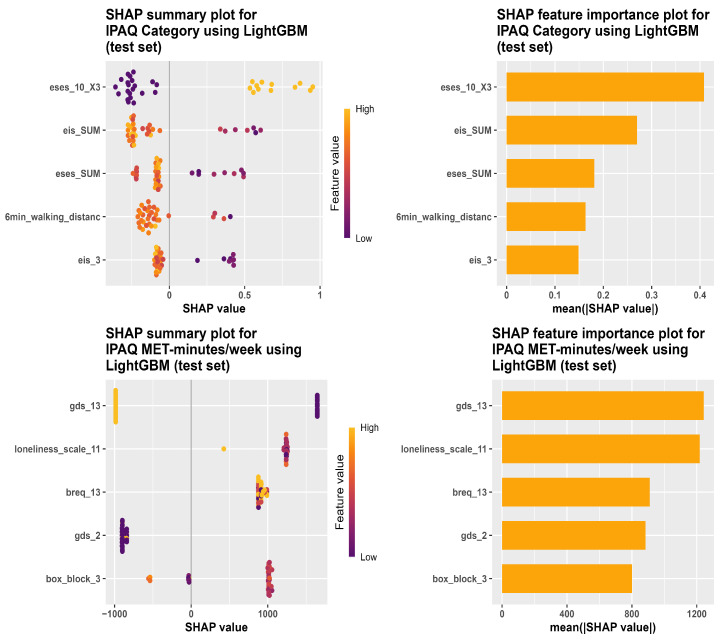
SHAP summary plots (**left**) and feature importance plots (**right**) for LightGBM models on the test set. In the SHAP summary plots, each dot represents one participant, the color indicates the feature value (low to high), and the x-axis shows the SHAP value. The top row shows the results for the IPAQ category, and the bottom row shows the results for IPAQ MET-minutes/week.

Figure 2 and Figure 3 show the most important predictors for several outcome variables based on the best-performing models selected from the previous analyses. The LightGBM variable importance scores were based on SHAP values, and predictors were ranked according to their mean absolute SHAP values.

### 3.2. Longitudinal Analysis

**Figure A3:**
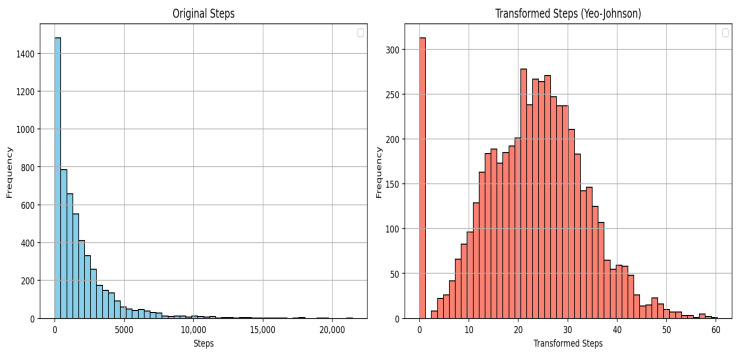
Outcome transformation using Yeo-Johnson transformation. Blue bars show original step counts and red bars show transformed step counts.

The outcome of interest in the longitudinal dataset was the number of steps (Steps), with a median of 1143 steps per time-of-day period [P25 = 375; P75 = 2374], and it ranged between 0 and 21,459 steps. The distribution of the number of steps was strongly right-skewed, with a large number of zero values and fewer observations with high step counts, as shown in Figure A3, which displays the distribution of step count (Steps) before and after applying the Yeo-Johnson transformation. The transformed values show considerably less skewness compared to the original data. The detailed model architectures and parameter settings are provided in Appendix A.6.

**Figure 4:**
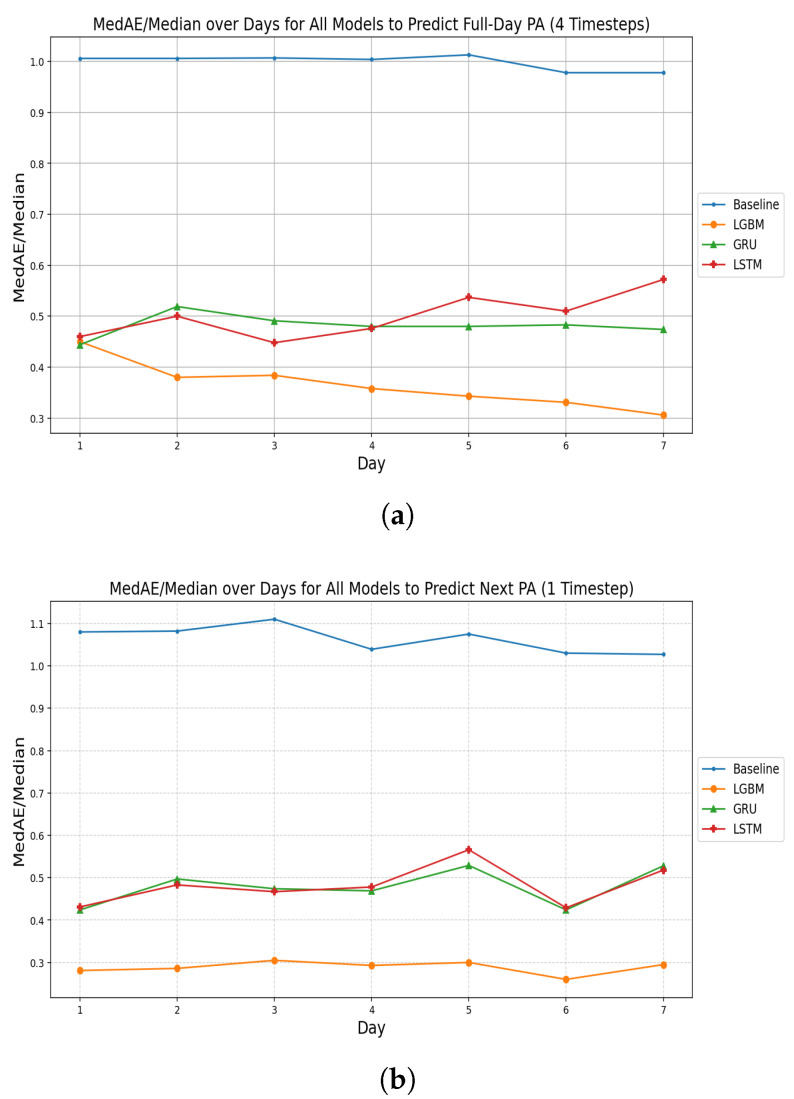
Model performance comparison across forecasting tasks. The x-axis shows the input sequence length (in days, 4 timesteps per day), and the y-axis shows prediction performance measured as MedAE/Median. Lower values indicate better predictive performance. (**a**) MedAE/Median across days for different models predicting next day step counts using a 4 timestep input window. (**b**) MedAE/Median across days for different models predicting short-term PA at the next timestep (1 timestep forecast).

Figure 4a shows the model comparisons to predict the number of steps for the entire next day (four timesteps). The blue line represents the baseline performance, which predicts the next step count by simply using the current step count. This approach did not involve any modeling and was included only as a reference point for comparing the performance of the developed models. All three models outperformed this baseline.

The results of the model comparisons indicate that the LightGBM model achieved the best performance, with the lowest MedAE/Median error ratios across days two to seven. Its error decreased gradually over the seven days, reaching a minimum of 0.31 on day seven. The GRU model showed moderate performance, with error values ranging from approximately 0.44 to 0.52. As for the LSTM model, it showed fluctuation in error ratios across the days compared to the GRU model. The LightGBM model, using step counts from the past seven days, was selected to forecast step counts for the next four timesteps due to its superior performance.

Figure 4b illustrates the model comparisons to predict the following step counts for a single timestep. The results showed that the LightGBM model consistently achieved low MedAE/Median error ratios between 0.26 on day six and 0.31 on day three, maintaining stable performance across the days and showing low sensitivity to input sequence length. The GRU model exhibited error values from approximately 0.42 to 0.53. The LSTM model showed decreased errors on day one and day six (about 0.46) compared to the other days.

Table 4 presents the performance of the LightGBM model for forecasting a full day and a single timestep ahead. For the full-day forecast, the model achieved a MedAE of 414.370 steps and a MedAE/Median ratio of 0.306. For the single-timestep forecast, the model achieved a MedAE of 345.930 steps and a MedAE/Median ratio of 0.260.

**Figure 5:**
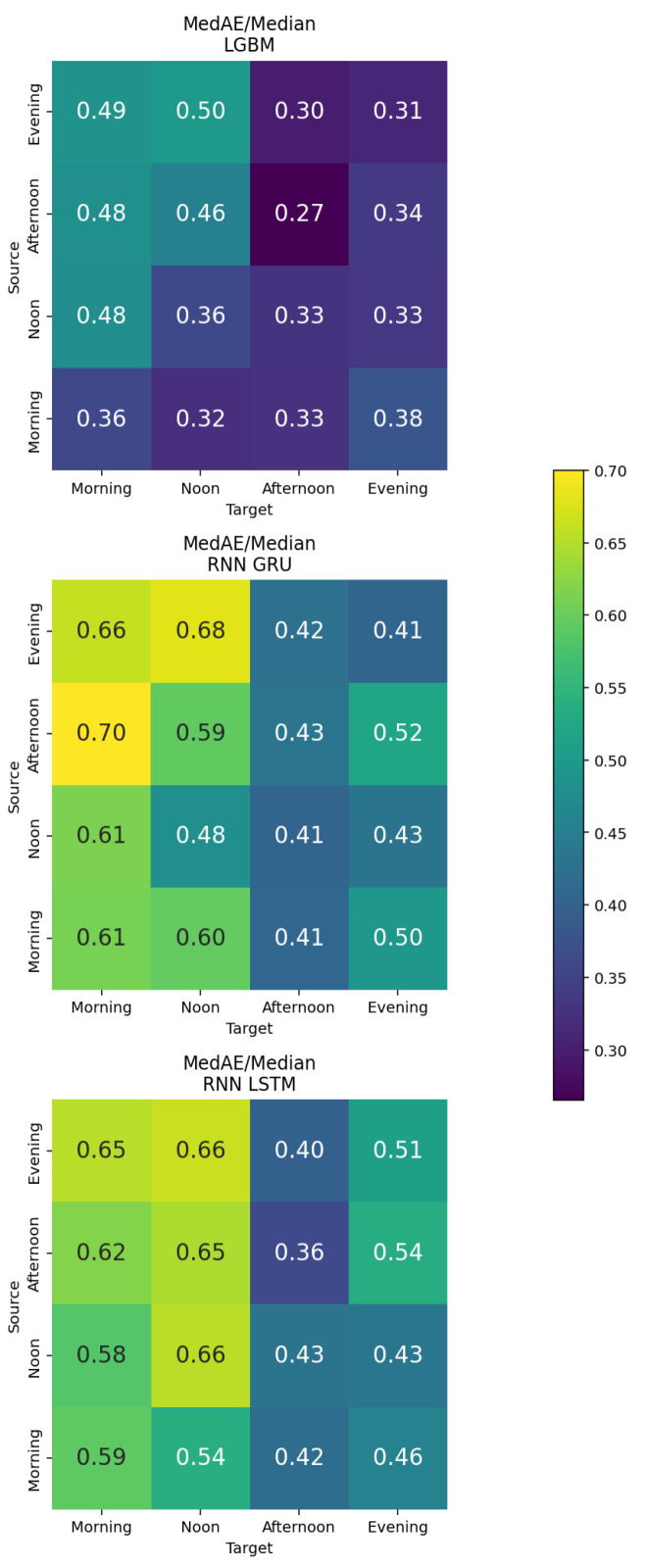
MedAE/Median for step-count forecasting across time-of-day segments, comparing LightGBM, GRU, and LSTM, where rows indicate the input segment (source) and columns the predicted segment (target). Lower values indicate better performance.

To further examine the model behavior, an additional analysis was performed using a fixed sequence length of six days with different temporal input–target arrangements. Instead of using sequences covering the entire day, each input consisted of step counts from a specific time segment (morning, noon, afternoon, or evening) across six consecutive days. The target was either the same or a different time segment on the next day. This approach was used to evaluate whether specific time-of-day combinations provided stronger predictive information for step count. The results are shown in Figure 5.

The LightGBM model trained on lagged step count features achieved the lowest overall MedAE/Median ratios. The best performance (0.27) was obtained for the afternoon-to-afternoon configuration, followed by evening-to-evening (0.31), and noon-to-noon and morning-to-morning predictions (0.36). These findings indicate that activity patterns in the afternoon were the most predictable across days.

For the GRU models, overall errors were higher compared to LightGBM. The best configurations were evening-to-evening (0.41) and morning-to-afternoon (0.41). The LSTM model showed similar behavior, with its lowest error (0.36) observed for the afternoon-to-afternoon configuration, outperforming the GRU model for the same time segment.

Overall, all models demonstrated lower performance when predicting across different time segments compared to within-segment forecasts. This suggests that time-specific step patterns were more informative than cross-segment relationships.

**Figure 6:**
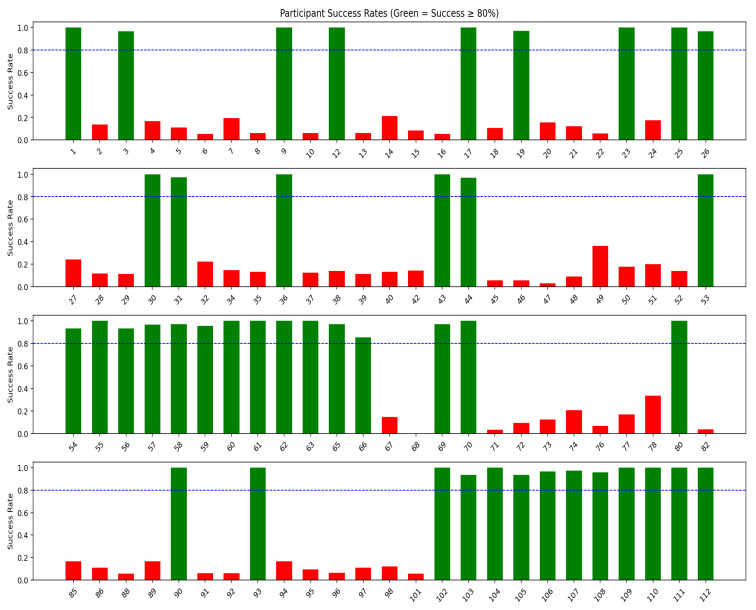
Per-participant success rates, defined as the proportion of predictions with proportion error ≤ 0.10. The x-axis shows participant ID, and the y-axis shows success rate. Each bar represents an individual participant. Green bars indicate participants who met the predefined success criterion (success rate ≥ 0.80), while red bars indicate participants who did not meet this threshold. The dashed horizontal line indicates the success criterion (success rate = 0.80).

Furthermore, a Leave-One-participant-Out (LOO) was conducted using a six-day input to predict the next single step count using the LightGBM model. The testing procedure involved iteratively holding out the data from one participant as the test set, while training the model on the data from the other 99 participants using the parameters in Table A1. This process was repeated for each participant in the whole dataset, so that every individual’s data was used once as a test set. The error was calculated separately for each participant’s prediction, based on the model trained without their data. Figure 6 shows the per-participant success rates for the following single step count predictions using the LightGBM model inputs and a six-day input sequence. Out of the 100 participants, 43 met the success criterion (shown in green bars).

To assess whether there was a systematic difference between participants in meeting the success criterion, different tests were conducted. These include the Wilcoxon rank-sum test for age and IPAQ as a continuous measurement, and Fisher’s exact test for the other variables. Table 5 presents the *p*-values from these tests. No comparisons were statistically significant, indicating no evidence of systematic differences based on the measured characteristics.

## 4. Discussion

The general aim of this study was to investigate different machine learning and deep learning methods and to identify the models that best predict PA, following two main objectives. The first objective was to identify key predictors associated with PA and related outcomes, including mild depression status and risk of falling. The second objective aimed to determine the optimal window size of previous step counts needed to accurately forecast PA. The main findings are discussed in relation to these objectives, followed by limitations and future directions.

### 4.1. Objective 1: The Cross-Sectional Analysis

In the cross-sectional analysis of mild depression status, self-reported PA item (IPAQ) emerged as the strongest predictor, suggesting that participants’ reported engagement in PA can capture differences related to reported mild depression. This is consistent with a previous study, which found that decreased PA measured using IPAQ among older adults tended to have higher GDS depressive symptom scores [38]. This association can be partly attributed to exercise-related neuromolecular changes, including higher Brain-Derived Neurotrophic Factor (BDNF) expression, and increased levels of serotonin and norepinephrine, which may alleviate depression [39]. This highlights the importance of promoting PA as a potential approach to prevent and reduce depression among older adults [38].

In contrast to the findings in this study, a study by [40] developed a LightGBM model to predict depressive symptoms measured with the Center for Epidemiological Studies-Depression Scale (CESD-10). It achieved an ROC AUC of 0.74, with self-rated health and nighttime sleep duration being the most important predictors. The differences in the predictors identified can be due to the difference between the depression scales, since the GDS focuses on psychological symptoms rather than somatic ones, which may lead to differences in how depression symptoms are captured and classified [41]. In addition, differences between the Chinese and European samples may partly reflect country-level context, as depression shows cross-national differences that may be attributed to differences in social environments [42]. Moreover, differences in the identified predictors may reflect differences in the candidate predictor set and feature-selection approach. Ref. [40] selected 16 variables as model input, whereas this study considered a larger number of features during feature selection prior to model training [40]. These differences indicate the importance of evaluating models within the intended context and outcome definition because predictive factors may not necessarily generalize uniformly across measurement scales and countries, highlighting the need for tailored validation in specific settings.

The SHAP analysis of the risk of fall prediction using LightGBM highlighted that the loneliness scale is a predictor of falling in older adults. This aligns with a previous study by  [43], which showed that loneliness is associated with an elevated risk of falls among older adults. A possible explanation is that loneliness can produce neuropsychiatric, neuromuscular, and other physiological changes, which can influence the risk of falling, although the direct mechanism is not fully confirmed [43].

Contrary to the findings reported in this study, ref. [44] developed several machine learning classification models for falling prediction using posturographic data from 215 community-dwelling older adults. For classification based on falling history in the previous year, they employed ensemble classifiers, and the models achieved an ROC AUC of approximately 0.70.

Unlike Liang et al. [44] who identified posturographic factors as the most important predictors of falling risk, the LightGBM model in this study did not find any balance control-related variables to be significant predictors. This is because the balance-related variables in this cross-sectional data were limited to clinical general balance tests, which may not provide strong predictive ability to classify participants who experienced a fall from those who did not. Liang et al. [44] observed that posturographic variables can be obtained across different stance tasks to distinguish how different sensory information supports stability and to characterize different components of postural control. This highlights the task specificity of these measures. In addition, they also noted that the predictive ability of the posturographic parameters was affected by the nature of the outcome of falling, with these parameters being less predictive when using previous falling history instead of the Timed-Up-and-Go (TUG)-based criterion as the outcome of interest.

When predicting the PA levels, exercise self-efficacy (ESES) was the most important factor identified by the LightGBM model. ESES is a person’s confidence to organize and do physical activities according to their own choice [45]. Within the COM-B (Capability, Opportunity, Motivation, and Behaviour) framework [46], ESES reflects motivation, because it captures if an individual is confident to carry out a physical exercise under difficult or challenging conditions. This may be because participants with higher ESES scores are more likely to participate in PA, and do not stop when difficulties are present, resulting in higher reported PA. From a Behavior Change Technique (BCT) viewpoint [47], the fact that ESES emerged as the most important predictor suggests that interventions targeting self-efficacy may help support PA in older adults. In BCT Taxonomy v1, the self-belief grouping includes methods such as verbal encouragement to increase self-efficacy, self-talk, emphasizing previous successes, and rehearsal of successful performance. These are developed to reinforce a person’s confidence to perform a PA behavior when challenges occur. Such findings are in line with a previous study showing that self-efficacy is associated with PA [48].

As for the IPAQ MET-minutes/week predicted using the LightGBM model, the most predictive factor influencing this outcome was a mild depression (GDS) item. This predictor of GDS reflects motivation in the COM-B framework, since automatic motivation includes mood disorders such as depression [47,49]. Depression can negatively affect PA in older adults by contributing somatic symptoms like fatigue, sleep disturbance, and delayed motor response [50].

Overall, the analysis revealed candidate predictors for PA and related outcomes, which can guide future work aimed at developing and targeting interventions to promote PA.

### 4.2. Objective 2: The Longitudinal Analysis

To address the second objective of this study, the LightGBM model using lagged step counts was selected due to its consistently superior performance compared to the RNN models. When forecasting PA for a full day, a sequence length of seven days (28 timesteps) yielded the best results. Similarly, when predicting the number of steps at a single future time point, a six-day window provided the best performance.

A seven-day input window likely provides an optimal balance between capturing stable habitual activity patterns and minimizing the influence of short-term variability in physical activity. In older adults, physical activity often follows weekly routines shaped by recurring behaviors, such as planned activities, appointments, and access to services, which tend to repeat across weekdays [51,52]. Incorporating several consecutive days of step-count history therefore allows the model to learn these cyclical patterns while smoothing out transient fluctuations related to weather, fatigue, or incidental events. This interpretation is consistent with our empirical results, which showed a gradual improvement in forecasting performance as the input window increased up to seven days, suggesting that shorter windows may not sufficiently capture weekly structure in activity behavior. Similar weekly regularities in physical activity among older adults have been reported in previous studies, supporting the relevance of a one-week temporal context for short-term PA forecasting.

Mamun et al. [18] conducted a study utilizing data collected from Fitbit Charge 2 wearable devices and the smartphone applications BeWell24 and SleepWell24. Their study included 99 participants, many of whom had more than 100 days of recorded observations. The authors employed LSTM models with a window size of seven days to predict the next day’s PA, measured as total daily step counts. They used multimodal features combining daily app engagement metrics, such as minutes used and times opened, along with PA measures, including sedentary duration, total device wear time, and other features. The final LSTM model achieved an MAE of 1677 steps for the prediabetic dataset and 2152 steps for the sleep dataset when forecasting next-day step counts.

In contrast to Mamun et al. [18], this study predicted PA using step counts divided into four three-hour time segments per day, rather than total daily step counts. The final model developed here used data from a seven-day window and relied only on step counts and time-of-day as inputs. This model achieved an MAE of 981 steps and a MedAE of 414 steps when forecasting next-day activity across the four time segments.

In this study, dividing step counts into four three-hour segments provides a more detailed description of daily activity than a single total step count. This approach can help identify time-of-day patterns and enables more specific next day predictions for particular periods of the day, which may be hard to predict when PA is summarized as daily total step counts.

With regard to the model combinations using fixed sequence lengths of six days for specific time segments, the analysis revealed notable differences in predictive performance depending on the input–target temporal alignment. The LightGBM model using lagged step counts achieved the best performance for within-segment predictions, specifically for afternoon-to-afternoon and evening-to-evening forecasts, which indicates that PA is more temporally consistent within the same time periods across days compared to across different periods of the day. Cross-segment configurations showed that forecasting morning targets was particularly challenging, especially from noon, afternoon, or evening PA, suggesting that morning PA may be less predictable by previous days’ activity in later segments and may be influenced by environmental or other factors. In contrast, afternoon and evening targets were less difficult to forecast. This pattern implies that specific time segments can be predictive of future PA using step count history alone, while other time segments like morning activity may need to incorporate contextual information that helps to capture different PA patterns.

The LOO analysis showed that for 43% of participants, the LightGBM model achieved a success rate of at least 80% when forecasting a single timestep. However, success rates were lower for the other participants. This difference in predictive performance across participants suggests that a single global forecasting model approach may be adequate for a subset of individuals who share a common PA behavior, but may not generalize equally well to others. For these individuals, using only previous step counts may provide limited information for modelling their PA patterns. These differences may reflect greater variability or irregularity in daily activity patterns. This may make forecasting more challenging for these participants.

An additional analysis was performed to determine whether participants who met the success criterion of having correct predictions differed from those who did not, based on demographic or clinical variables such as age, gender, falling risk, IPAQ category, and mild depression status. The results showed no statistically significant differences, indicating that variations in predictive performance were not systematically linked to these factors. This suggests that other unmeasured factors may contribute to performance differences across participants. These findings highlight that while a global model provides a strong baseline and performs well for a substantial proportion of participants, prediction accuracy may vary across individuals. Therefore, some older adults may benefit from personalised models or additional predictors to further improve PA forecasting performance.

Overall, this longitudinal analysis demonstrates that step-count history alone can support meaningful short-horizon forecasting of physical activity in community-dwelling older adults. By modelling activity at the level of time-of-day segments, the approach provides a practical way to anticipate when activity is likely to be lower or higher on the next day. This could inform more timely and targeted interventions. In addition, the observed differences across time segments suggest that some periods of the day provide more stable and informative signals for forecasting than others. This insight may help inform the selection of time periods for future monitoring and model development.

### 4.3. Limitations and Drawbacks of the Methods

In the cross-sectional analysis, the fall-risk prediction model demonstrated limited discriminative performance, as reflected by a low PR AUC (0.381). This suboptimal performance is primarily attributable to the pronounced class imbalance inherent to fall-risk datasets and the restricted set of balance-related features available in the current study. While we evaluated several commonly used mitigation strategies, including resampling techniques and class weighting, these approaches resulted in only marginal or negligible improvements in PR AUC and did not yield a model suitable for reliable clinical prediction. These findings suggest that standard imbalance-handling methods may be insufficient when fall events are rare and signal strength in the predictors is limited. Future work should therefore prioritize the integration of richer and more diverse balance and mobility features, potentially derived from longer monitoring periods or multimodal sensor data. In addition, alternative modeling paradigms such as anomaly or novelty detection may be more appropriate for this context, as they frame falls as rare deviations from an individual’s typical movement patterns rather than as a conventional binary classification problem. Such approaches may offer improved sensitivity to subtle precursors of falls and warrant further investigation in longitudinal and higher-resolution datasets. Other methodological limitations should be considered when interpreting the findings of this study. First, although the cross-sectional dataset initially included a large number of candidate predictors relative to the sample size, multiple safeguards were implemented to reduce the risk of overfitting. These included the removal of near-zero variance features, correlation-based filtering, and information-gain ranking to retain only the most informative predictors prior to model training, as well as the use of regularized models such as Elastic Net and constrained tree-based boosting. Nevertheless, given the limited sample size, some residual risk of overfitting—particularly for flexible models such as LightGBM—cannot be entirely excluded and should be acknowledged when interpreting the cross-sectional results. In contrast, the longitudinal analysis did not involve high-dimensional predictors but several limitations should also be considered when interpreting the findings of this part. First, the comparison of forecasting models was limited to LightGBM and two widely used recurrent neural network architectures (LSTM and GRU). While these models represent established baselines in time-series forecasting and digital phenotyping research, more recent architectures—such as attention-based or transformer-based models—were not evaluated. The exclusion of such models reflects the relatively small sample size and short monitoring duration (14 days), which may be insufficient to effectively train more data-hungry architectures without risking overfitting. Second, the longitudinal models relied exclusively on historical step-count data segmented by time-of-day, without incorporating contextual or behavioral variables (e.g., weather, sleep, health events, or environmental factors) that may influence daily physical activity. This reliance on step-count history alone likely contributed to the observed participant-specific variability in prediction performance, as only 43% of participants met the predefined success criterion in the leave-one-participant-out analysis. Third, participants with substantial missing data were excluded, which may have introduced selection bias toward individuals with more regular device use and more stable activity patterns. Finally, the use of a single global model assumes homogeneity in activity dynamics across participants, which may not hold for individuals with irregular or highly variable routines. Together, these limitations indicate that while the proposed approach supports meaningful short-term physical activity forecasting for a subset of older adults, future studies with longer follow-up periods, larger datasets, and richer contextual information should explore more advanced architectures and personalized modeling strategies to further improve generalizability and robustness.

### 4.4. Ideas for Future Work and Research

Future work should include collecting more data (increasing the number of participants and other types of data that could influence the level of physical activity, such as weather or sleep variables) for both the cross-sectional and longitudinal datasets. Having larger and more diverse data can help improve the generalizability of the predictive models and allow for a better understanding of the variables that serve as reliable predictors. This increased data availability may also support capturing a wider range of PA patterns and behaviors, helping the models to generalize better across diverse populations. Given the low prevalence of falls and the retrospective nature of the outcome, future studies may benefit from reframing fall risk prediction as a rare-event or anomaly detection problem, particularly when richer longitudinal sensor data are available.

Nevertheless, the methodological framework developed in this study is intended to be extended and validated in larger, richer datasets, with additional predictors to assess generalizability.

## 5. Conclusions

This study was exploratory and predictive in nature, aiming to assess modeling feasibility and performance. We examined the application of machine learning and deep learning techniques to predict physical activity levels in older adults, using both cross-sectional and longitudinal datasets. Several models were evaluated, including Linear and Logistic Regression, LightGBM, RNNs such as GRU and LSTM, and Elastic Net.

In the cross-sectional analysis, models were developed to predict PA levels and related outcomes such as falling risk and mild depression status. The LightGBM model achieved the best overall performance. The most important predictor identified for the IPAQ category outcome was an item from the ESES, indicating that specific aspects of exercise self-efficacy play a key role in distinguishing between high and moderate physical activity levels.

In the longitudinal analysis, time series models were trained to predict step counts using sequences of past observations. The results show that a seven-day input sequence provided the best predictive performance for full-day PA, while a six-day window was optimal for single-time-step forecasts. However, model performance varied across individuals.

Rather than identifying a universally superior algorithm, the findings highlight that data structure, temporal context, and feature relevance are more critical determinants of predictive performance than model complexity. This study demonstrates the potential of combining wearable sensor data and machine learning methods to better understand and predict physical activity in older adults. Using only previous step counts, the model predicted next-day physical activity accurately for a subset of participants, while others showed more variable patterns that may require additional contextual predictors to improve forecasting. Once richer datasets are collected, the frameworks developed in this study could be augmented and used to support personalized healthcare monitoring and interventions for older adults.

## Figures and Tables

**Table 1:** Cross-sectional data summary statistics. Continuous data are presented as mean (SD) or median [P25; P75] according to the distribution of the data. The outcome variables are IPAQ MET-minutes/week, IPAQ category, GDS category, and falling in the past 6 months.

Continuous Variable	Statistic	Minimum–Maximum
Age (years)	70.10 (4.59)	64–87
BMI (kg/m^2^)	26.30 [23.00; 28.40]	19.00–42.30
6 min walking distance test	572.40 (90.80)	240–855
Speed	5.91 (0.80)	3.80–8.40
WHOQOL Physical Health	76.00 (11.80)	39.29–100.00
WHOQOL Psychological	72.30 (10.20)	45.83–91.67
WHOQOL Social	75.00 [66.70; 83.30]	25.00–100.00
WHOQOL Environment	83.70 (10.10)	56.25–100.00
IPAQ MET-minutes/week	5143.50 [2642; 9973.30]	99–64,848
**Categorical Variable**	**Value**	**n (%)**
Sex	Male	47 (43.52%)
	Female	60 (55.55%)
	Other	1 (0.93%)
Marital state	Single	8 (7.40%)
	Living together	9 (8.30%)
	Married	78 (72.20%)
	Divorced	8 (7.40%)
	Widow	5 (4.60%)
Physical constraints	Yes	8 (7.40%)
Retired	Yes	105 (97.20%)
Living situation	Living with partner	85 (78.70%)
	Living alone	20 (18.50%)
	Living with children	1 (0.90%)
	Other	2 (1.90%)
IPAQ category	Low	1 (0.90%)
	Moderate	30 (27.80%)
	High	77 (71.30%)
GDS category	Mild depressed	36 (33.30%)
Falling in the past 6 months	Yes	18 (16.70%)

SD = standard deviation, P25 = 25th percentile, P75 = 75th percentile, BMI = Body Mass Index, WHOQOL = World Health Organization Quality of Life, IPAQ = International Physical Activity Questionnaire, MET = Metabolic Equivalents of Task, GDS = Geriatric Depression Scale.

**Table 2:** Evaluation metrics for binary outcomes.

Metric	GDS			Fall			IPAQ		
	LR	EN	LGBM	LR	EN	LGBM	LR	EN	LGBM
F1 Score	0.615	0.476	0.571	0.353	0.300	0.267	0.303	0.682	0.840
Precision	0.533	0.500	0.600	0.273	0.214	0.222	0.556	0.750	0.808
Recall (Sensitivity)	0.727	0.455	0.545	0.500	0.500	0.333	0.208	0.625	0.875
Specificity	0.682	0.773	0.818	0.714	0.607	0.750	0.556	0.444	0.444
Accuracy	0.697	0.667	0.727	0.676	0.588	0.676	0.303	0.576	0.758
Balanced Accuracy	0.705	0.614	0.682	0.607	0.554	0.542	0.382	0.535	0.660
PR_AUC	0.444	0.504	0.800	0.174	0.190	0.381	0.653	0.764	0.809

LR = Logistic Regression, EN = Elastic Net, LGBM = LightGBM.

**Table 3:** Evaluation metrics for IPAQ MET minutes/week.

Model	MAE	MedAE	MAE/Mean	MedAE/Median
LR	7349	4439	0.801	0.859
EN	6049	3974	0.704	0.785
LGBM	6102	2788	0.711	0.551

LR = Logistic Regression, EN = Elastic Net, LGBM = LightGBM.

**Table 4:** LightGBM model evaluation metrics on the test set for full-day (left, using seven days of input) and single-timestep (right, using six days of input) forecasting.

Full-Day Forecast		Single-Timestep Forecast	
Metric	Value	Metric	Value
MAE	981.150	MAE	933.570
MedAE	414.370	MedAE	345.930
Mean	2083.780	Mean	2041.320
Median	1355.000	Median	1330.000
MAE/Mean	0.471	MAE/Mean	0.457
MedAE/Median	0.306	MedAE/Median	0.260

**Table 5:** *p*-values from Wilcoxon and Fisher’s exact tests examining differences in participant characteristics between those meeting and not meeting the success criterion.

Variable	*p*-Value
Age	0.803
IPAQ category	0.366
Sex	1.000
Falling risk	0.598
GDS category	0.668
IPAQ MET-minutes/week	0.549

**Figure A1:**
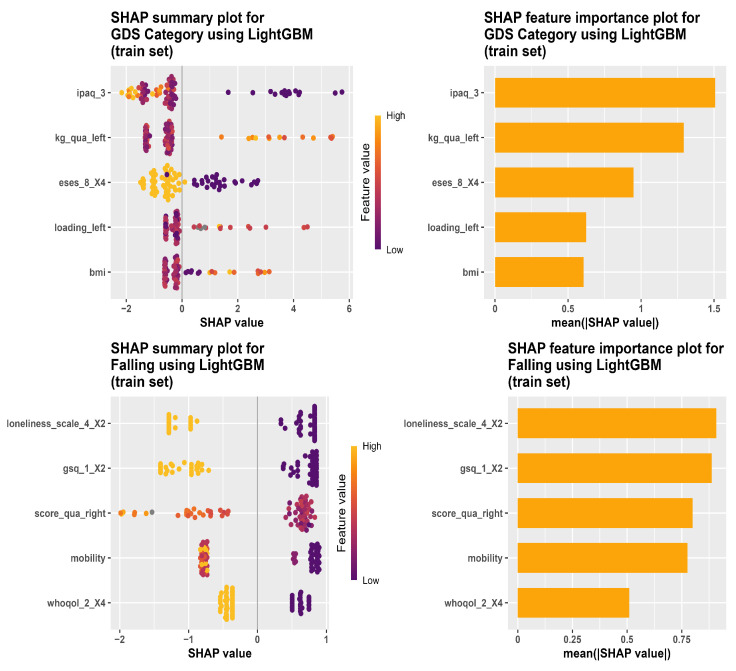
SHAP summary plots (**left**) and feature importance plots (**right**) for LightGBM models on the train set. In the SHAP summary plots, each dot represents one participant, the color indicates the feature value (low to high), and the x-axis shows the SHAP value. The top row shows the results for the GDS category, and the bottom row shows the results for falling.

**Figure A2:**
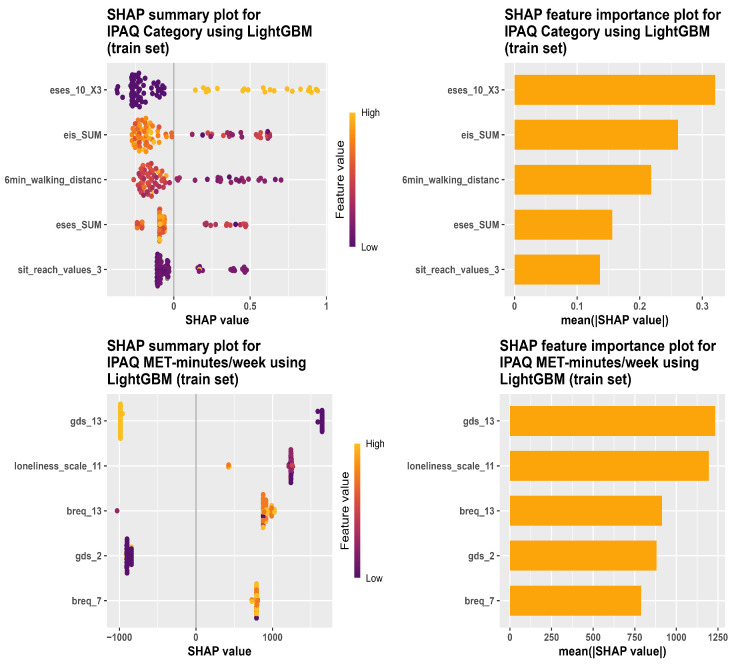
SHAP summary plots (**left**) and feature importance plots (**right**) for LightGBM models on the train set. In the SHAP summary plots, each dot represents one participant, the color indicates the feature value (low to high), and the x-axis shows the SHAP value. The top row shows the results for the IPAQ category, and the bottom row shows the results for IPAQ MET-minutes/week.

**Figure A4:**
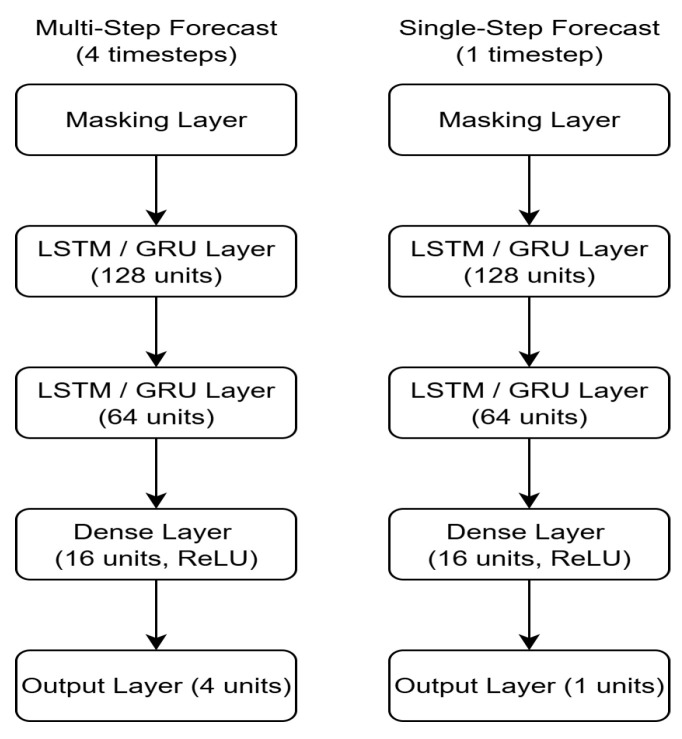
RNN model architectures.

## Data Availability

The data presented in this study are available on request from the corresponding author due to privacy, legal or ethical reasons.

## Appendix A

### Appendix A.1. Ethics

The study was registered at https://clinicaltrials.gov/study/NCT06094374 (NCT06094374) on 17 October 2023 and approved by the Ethical Committee of Hasselt University (B1152023000011). The full study protocol detailing recruitment strategies, data collection procedures, and analytical methods has been presented separately [20]. Informed consent was obtained from all subjects before participation.

The data used were anonymized before being shared with the author. Both the cross-sectional and longitudinal datasets were shared under ethical and institutional approval. The longitudinal data, which were collected using Garmin devices, were approved by the Ethical Committee at Hasselt University [20].

### Appendix A.2. Data Preprocessing

When processing the cross-sectional data, variables were categorized based on the number of unique values. Specifically, variables with five or fewer unique values were treated as categorical, and they were dummy-coded before model training. In contrast, variables with six or more unique values were regarded as continuous and were treated as numerical predictors for model training. All cross-sectional analyses were conducted using R version 4.3.3.

To handle missing values in some features in the cross-sectional dataset, multiple imputations using the mice package in R were performed. The method of imputation relied on the distribution of different variables [53]. For categorical variables with more than two unique values, Proportional Odds Logistic Regression (polr) was used. Logistic Regression (logreg) was utilized to impute the binary variables, and Predictive Mean Matching (pmm) was used to impute the continuous variables. Ten imputations were conducted with ten iterations to generate ten complete datasets. For models that relied on the imputed datasets, such as Logistic Regression and Elastic Net, each complete dataset had its own coefficients, which were then used to generate the predictions on the test data, producing ten predicted values. These predictions were then averaged to obtain the final predicted value from the test set. The longitudinal analyses were performed using Python version 3.10.18.

### Appendix A.3. Supplementary Methods and SHAP Results for the Cross-Sectional Analysis

The features in the cross-sectional data were normalized using the mean and standard deviation of the training set before model training. For the logistic regression and linear regression models, a step-up approach based on Akaike Information Criterion (AIC) was used for model selection. To tune the parameters in the cross-sectional analysis, Bayesian optimization was employed, as it is more efficient than the full grid search approach and typically yields better optimized parameters than random search [54]. Class weights were applied during training to address class imbalance (especially for the fall risk and IPAQ category) and improve the model’s ability to identify minority class observations [55].

Figure A1 and Figure A2 show the SHAP plots for the train data in the cross-sectional analysis.

### Appendix A.4. Supplementary Methods for the Longitudinal Analysis

To improve the training and performance of the regression models, the outcome variable in the longitudinal analysis was transformed using the Yeo-Johnson transformation, which helps to reduce skewness in highly skewed data [56]. This transformed outcome was used during the model training process. After obtaining predictions from the models, the values were converted back to the original scale by applying the inverse transformation.

### Appendix A.5. Transformation

This figure illustrates the distribution of step counts before and after applying the Yeo–Johnson transformation.

### Appendix A.6. Model Specifications

This section provides the technical details of the models used in the longitudinal analysis, including the architecture of the RNN models and the parameter settings applied for the LightGBM models. Training of the GRU and LSTM models was conducted using 20 epochs, a batch size of 16, and a learning rate of 0.005. The model architectures consisted of the layers illustrated in Figure A4, while the parameter values used for the LightGBM models are summarized in Table A1. The median and interquartile range (IQR) of the training set were used for normalization of the features due to their skewed distribution. The deep learning models were trained using the Adam optimizer and a loss function of MedAE.

**Table A1:** LightGBM parameters.

Parameter	Value
Number of trees	3000
Maximum leaves per tree	1000
Maximum tree depth	100
Minimum child samples	1
Minimum split gain	0
Subsample fraction	1
Learning rate	0.005
L1 regularization	0.01
L2 regularization	0.01
